# State of the Art in Incremental Forming: Process Variants, Tooling, Industrial Applications for Complex Part Manufacturing and Sustainability of the Process

**DOI:** 10.3390/ma17235811

**Published:** 2024-11-27

**Authors:** Gabriela-Petruța Popp, Sever-Gabriel Racz, Radu-Eugen Breaz, Valentin Ștefan Oleksik, Mihai-Octavian Popp, Dana-Elena Morar, Anca-Lucia Chicea, Ilie-Octavian Popp

**Affiliations:** 1 Department of Industrial Machines and Equipment, Engineering Faculty, Lucian Blaga University of Sibiu, 550025 Sibiu, Romania; gabriela.popp@ulbsibiu.ro (G.-P.P.); gabriel.racz@ulbsibiu.ro (S.-G.R.); valentin.oleksik@ulbsibiu.ro (V.Ș.O.); mihai.popp@ulbsibiu.ro (M.-O.P.); anca.chicea@ulbsibiu.ro (A.-L.C.); ilie.popp@ulbsibiu.ro (I.-O.P.); 2 Department of Civil Construction and Management, Faculty of Civil Engineering, Technical University of Cluj-Napoca, 400114 Cluj-Napoca, Romania; dana.morar@ccm.utcluj.ro

**Keywords:** incremental forming, state-of-the-art, single point incremental forming (SPIF), two point incremental forming (TPIF), conjugated active plate, complex part manufacturing, dimensional accuracy, surface quality, tooling, industrial applications

## Abstract

This paper explores the development and application of the incremental forming process, an innovative method for manufacturing complex parts with high flexibility and low tooling costs. The review categorizes three key process variants: Single Point Incremental Forming (SPIF), Two Point Incremental Forming (TPIF), and Incremental Forming with Conjugated Active Plate (IFCAP). This study demonstrates the significant effects of these process variants on part accuracy and material behavior, particularly under varying process conditions. This study identifies critical technological parameters such as tool diameter, feed rate, and vertical step size. The findings also demonstrate the role of optimized toolpaths and lubrication in improving process efficiency. Applications of incremental forming across various industries, including automotive, aerospace, medical, and construction, demonstrate its versatility in prototype production and small-series manufacturing. These results contribute to a deeper understanding of incremental forming, offering practical recommendations to enhance precision, scalability, and material formability, and supporting future innovations and broader industrial applications.

## 1. Introduction

**Figure 1:**
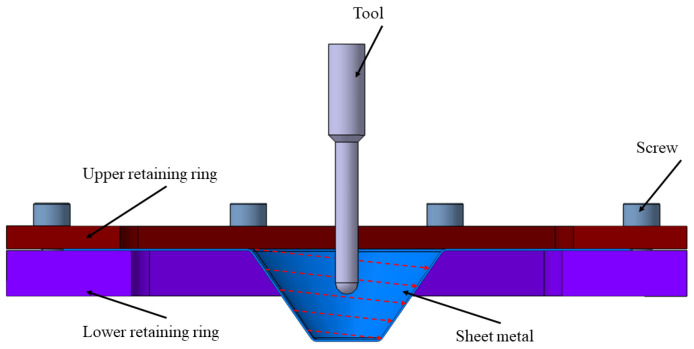
Principle of incremental forming.

The concept of incremental forming emerged in 1967 when Leszak obtained the patent for “Apparatus and Process for Incremental Dieless Forming” [1]. The incremental forming process has gained increasing popularity among researchers worldwide due to its high potential for manufacturing complex parts. The main advantages of the incremental forming process include the use of simple-shaped tools and the absence of a die that represents the conjugate shape of the part, with the sheet metal blank being clamped at the edges between the blank holder and the retaining ring. The operating principle of incremental forming is straightforward: a punch with a hemispherical or parabolic shape follows a specific toolpath, thereby deforming the sheet metal blank to obtain a part with a complex shape (Figure 1). Thus, the plastic deformation of the sheet occurs progressively until the final shape of the part is achieved [2]. In recent years, incremental forming has been applied in industries such as automotive, aerospace, and medical, particularly for prototyping and low-volume production of complex geometries. For instance, it has been used to create customized cranial implants in the medical field and lightweight panels in automotive and aerospace applications, showcasing its versatility and cost-effectiveness. However, incremental forming requires a longer processing time compared to the deep drawing process, which produces a part in a few tenths of a second. According to the article by Jeswiet and his collaborators, the incremental forming process is carried out using a numerically controlled machine tool or a robot that follows a specific toolpath to achieve the desired shape of the part [3].

The main benefits of the incremental forming process are:

- Ease of implementation—Three-axis CNC machine tools or industrial robots can easily perform this process;
- High flexibility—The quick and easy changing of toolpaths used in part manufacturing allows for the forming of a wide range of products with varying shapes and sizes;
- Low-cost tools—The costs for the punches used are low compared to those required for conventional processes;
- Short lead time—In conventional plastic deformation processes, the time to produce prototypes ranges from 8 to 25 weeks [4], but by using the same tools and merely altering their toolpath, it is possible to produce prototypes in just a few hours through the incremental forming process;
- High deformability—Compared to conventional plastic deformation processes, it is possible to achieve greater deformations in the studied process [3].

Unfortunately, this process is still in the early stages of development, and although considerable progress has been made in recent years, more research is needed to achieve parts with high shape and dimensional accuracy that meet industry standards. To be suitable for production, certain aspects of the process need to be regulated. In the following, we will provide an overview of the research conducted in recent years in the field of incremental forming up to this date. The novelty of this research lies in its comprehensive analysis of various incremental forming process variants, including Single Point Incremental Forming (SPIF), Two Point Incremental Forming (TPIF), and Incremental Forming with Conjugated Active Plate (IFCAP). By systematically comparing these methods, this study examines their distinct impacts on the incremental forming process, with a focus on deviations observed in the process. This research also presents recent experimental data, expanding existing knowledge and addressing previously unexplored aspects of incremental forming. The structure of the paper follows the development of these process variants, their technological parameters and applications, and presents future research directions, emphasizing areas that need further exploration to optimize the process for industrial-scale production.

## 2. Classification of the Incremental Forming Process

Research in the field conducted in recent years has revealed various methods for optimizing and improving the techniques used. The incremental forming process appears in a wide variety of configurations. The main criteria for classifying the incremental forming process are:

- based on the active forming element: with punch, with roller, with water jet, with laser, with ball jet;
- based on the active support element: without support element, with counterpunch, with roller, with mandrel, with active plate with partial or full dies;
- based on the method of force application: continuous or intermittent;
- based on the workpiece clamping system: fixed or mobile;
- based on temperature: hot or cold;
- based on the number of contact points between sheet metal and the tool: single point or multiple points.

**Figure 2:**
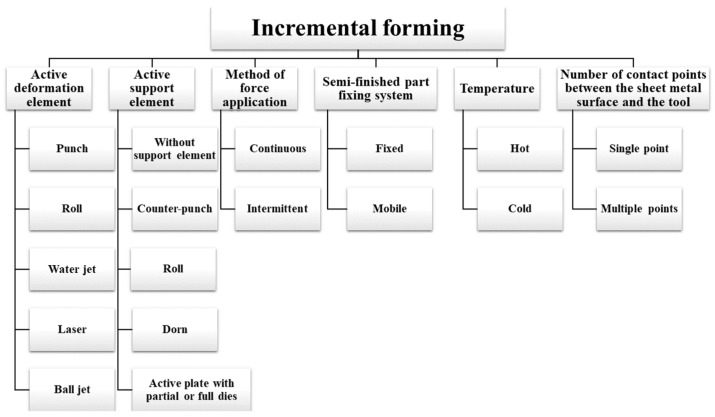
Variants of the incremental forming process.

A classification of the incremental forming process is shown in Figure 2.

The most recent and innovative variants based on forming tools can be classified as: Single Point Incremental Forming (SPIF), Incremental Forming with Conjugated Active Die (IFCAD), and Two Point Incremental Forming or with Counterpunch (TPIF). Table 1 summarizes the key characteristics, applications, advantages, and disadvantages of the main incremental forming variants, providing a comprehensive comparison to highlight their specific features and industrial relevance. These variants aim to improve the dimensional and shape accuracy of the parts obtained through incremental forming. The major difference between SPIF and IFCAP is the presence of an active plate or a punch in the latter variant, which acts as support for the material.

### 2.1. Single Point Incremental Forming Process

**Figure 3:**
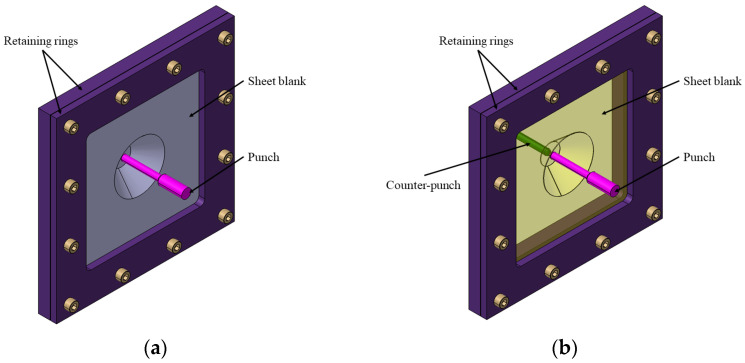
(**a**) Principle of the single-point incremental forming process; (**b**) Principle of the two point incremental forming process or with counterpunch.

The single-point incremental forming process is the simplest and most economical variant of this type of incremental forming. A sheet metal blank, with the edges fixed, is deformed by a spherical-, parabolic- or conical headed tool, following a predetermined path. The process is flexible, allowing various geometries to be achieved without expensive dies, making it ideal for small series production and prototyping. However, it has drawbacks such as long lead times and angle limitations. Optimization of parameters such as the rotational speed of the tool, feed rate, vertical step, and toolpath is essential to improve part quality and accuracy. Figure 3a illustrates the principle of the single point incremental forming process, highlighting the key components involved in this method.

Najm and Paniti have studied the influence of technological parameters such as the material from which the punch is made, the shape, the diameter, and the roughness of the punch [5].

Maqbool and Bambach studied the dimensional accuracy of parts manufactured by the single-point incremental forming process, as well as the mechanical phenomena occurring in the material during the process. They used an analytical model to determine how plastic deformations are distributed in the material when the forming process takes place. Results obtained by the analytical model have been validated by experiments, and the results show that both bending and shear occur in the material [2].

### 2.2. Two Point Incremental Forming Process or with Counterpunch

Initially, this variant was proposed in 2007 by Meier and collaborators in order to balance the forces applied in the incremental forming process for better control over the strain localization and to improve the deformability of the material and the accuracy of the formed parts [6]. In this process, a punch is used to deform the semifinished part, while another punch, namely the counterpunch, provides support to the semifinished part. Figure 3b shows the principle of this variant of the process. The toolpaths of the two punches are complementary and synchronized to take into account the wall angle of the deformed part and the thickness of the blank.

Meier and collaborators compared parts produced by the single-point incremental forming process with the counterpunch process. They deformed 0.6 mm thick aluminum alloy Al 99.5 and K16449388IF steel semifinished parts to a frustrum cone shape. The results showed that the material thickness distribution is much more homogeneous, and the parts are much more accurate in the TPIF process than in the SPIF process [6].

Wang and collaborators, in their work, studied the accuracy of 0.71 mm thick AA5052—O aluminum alloy parts deformed by SPIF and TPIF. TPIF implementation was performed on a CNC machine using a C-frame and a blank clamping system. It has been shown that, in terms of part accuracy, the two point incremental forming method provides 7% higher accuracy than the single-point incremental forming method. In the paper written by Meier and collaborators, a comparison between parts made of 0.5 mm thick AlMn99.8 1hh aluminum alloy deformed by SPIF and TPIF is presented. Both variants were performed using a KUKA KR 360 industrial robot from KUKA Robotics, Augsburg, Germany. From this comparison, an improvement of the part surface quality and a reduction in the forming forces was observed for the TPIF method [7].

### 2.3. Incremental Forming Process with Conjugated Active Die

**Figure 4:**
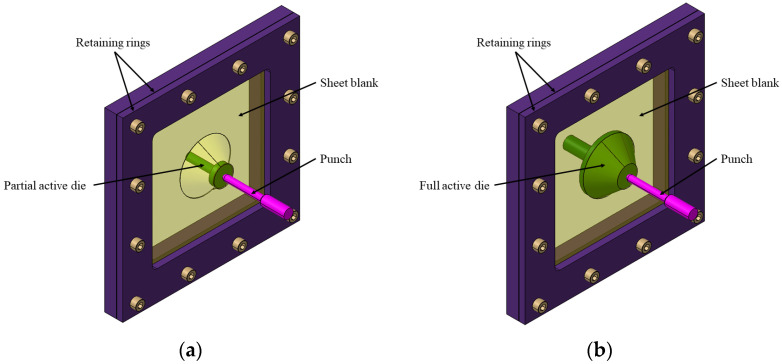
Principle of the incremental forming process with conjugate active die with (**a**) partial active die and (**b**) full active die.

The incremental forming process with a conjugated active die is similar to other methods where the sheet metal blank is fixed between the active die and the retaining ring. The punch follows a toolpath on the outer surface, deforming the part from tip to base. This process can be configured with a mobile punch and either a partial, full positive, or full negative active die that defines the part’s shape. The active die supports key areas of the blank (Figure 4) [8]. While this method improves accuracy, surface quality, and efficiency, its use of an active die reduces flexibility compared to SPIF.

This variant of the incremental forming process results in parts with good accuracy, as the sheet is deformed by the punch while being supported by the active die during the process [3]. From a cost perspective, this method is more expensive than SPIF due to the use of the active dies. The main drawback of this process is its reduced flexibility, as a different active die is required for each distinct shaped part [9]. The incremental forming process with conjugated active die is favored by the Japanese company Amino North America Corporation (St. Thomas, Ontario, Canada) because of the easier control it exerts over the deformability of the material. So, from an industrial point of view, it is of interest because of the good accuracy it gives despite the cost of using the active dies.

## 3. Technological Parameters Used in the Incremental Forming Process

### 3.1. Types of Equipments [Used in Incremental Forming Process

The equipment on which the incremental forming process can be performed has a significant influence on the incremental forming process because the equipment can influence the rotational speed and feed rate of the punch, the positioning accuracy, the complexity of the toolpath, the maximum forces allowed and the degrees of freedom of the punch. Leszak was the first researcher to present the equipment needed to accomplish this process [1].

Specialized tools for the incremental forming process are currently not widely available and are extremely expensive. The only company that produces such equipment is the Japanese company Amino, and they cost between 100,000 and 200,000 euros. However, most researchers in the field prefer to optimize their own equipment to perform the process. The types of equipment used are CNC machine tools or industrial robots.

**Figure 5:**
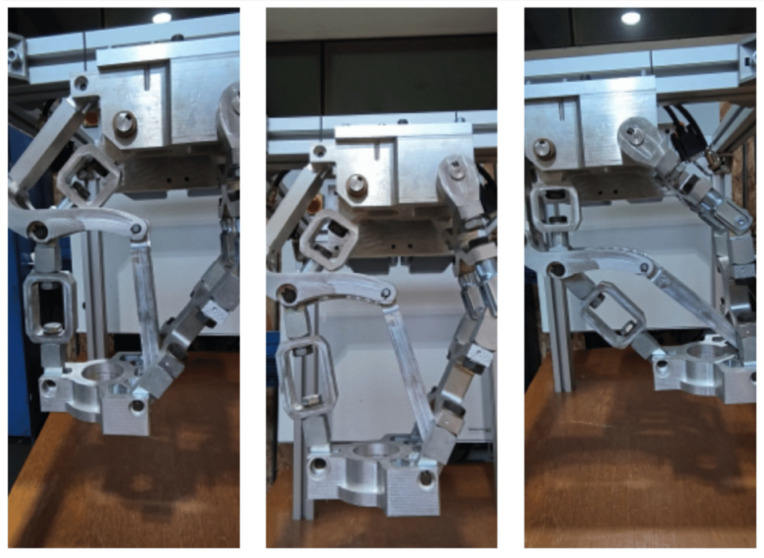
Machines built specifically for the incremental forming process [14].

Automation and specialized equipment are essential for increasing efficiency and precision in the incremental forming process [10]. Early designs, such as the rectangular frame proposed by Iseki [11], relied on manual operation, requiring human skills and hand tools to apply force incrementally. However, automated equipment is now recommended to ensure higher precision and repeatability. Thus, some researchers in the field have proposed several variants of equipment specifically designed to perform the incremental forming process, such as those developed by the Amino Corporation or other research groups. Recently, Amino has developed the “Space Former”, which utilizes an incremental forming process with conjugated active dies to provide a rigid support for the part. The largest piece of equipment designed by this company measures 6.5 × 2.5 m and accomplishes the incremental forming process in a single point. Allwood and its collaborators have developed a specialized platform at Cambridge University to perform the incremental forming process. This equipment has the ability to sustain the high forces required to deform the blanks and give the positioning accuracy required for this process. An active die can be used at the bottom of the blank to control the deformation of the blank. This equipment has been designed to withstand forces up to 13 kN in the vertical direction and 6.5 kN in the horizontal directions [12]. Rauch has designed a platform, called VERNE, whose principle of operation resembles that of the Stewart platform with the architecture of a parallel robot. This rig was designed to perform the same types of motions as a CNC machine tool [13]. Building upon these advancements, Resende et al. [14] have developed a compact incremental forming machine (Figure 5) that integrates advanced kinematics and control systems. This innovative design prioritizes portability and efficiency, offering a versatile solution for incremental forming processes.

Most researchers use CNC machine tools and industrial robots to perform the incremental forming process. In general, all 3-axis CNC machine tools are suitable for performing the studied process due to their rigidity and high productivity rate. The toolpath is generated using Computer Aided Manufacturing (CAM) software packages [15].

As an alternative to machines specifically designed for the incremental forming process, some researchers have used industrial robots. This solution has proven to be viable due to its high flexibility, having six or even more degrees of freedom and allowing much more convenient positioning of the punch on the sheet [16]. It also makes it possible to combine several production steps in a production cell with a single robot. Implementing the process with industrial robots is very similar to its implementation on CNC machine tools: a 3D model is imported into a CAM software, such as SprutCAM X, that generates the toolpaths for the punch.

In both cases, certain adaptations are necessary to perform the studied process. It is necessary to design a holder for the tool to withstand the forces that may occur during the deformation of the blanks, as well as fixing elements of the blanks to allow translational movements on its surface. Although CNCs and industrial robots are easily adaptable, they have some limitations that can arise during the process. In industrial robots in particular, serial kinematics led to a cumulative error from each joint which results in lower positional accuracy of end effectors. For these reasons, most experiments are performed on low strength materials [17].

### 3.2. Tools Used for Incremental Forming

Currently, the tools used in incremental forming are not yet standardized; as with other chipping processes, the punches used in this process must be designed and produced according to the requirements of each product. Choosing the right punch is an important factor in producing parts with the desired shape and without defects. Most researchers have used punches with a radius between 2 mm and 25 mm to study the influence of punch radius on the accuracy of incrementally deformed parts. Ham and Jeswiet demonstrated that the interaction between the punch diameter and material thickness influences the maximum achievable wall angle of the part [18].

Hussain and collaborators investigated the influence of some parameters on the deformability of AA-2024 blanks. The parameters chosen by them were punch diameter, vertical step, and feed rate. They showed that the vertical step size and the diameter of the punch have the greatest influence on the deformability of the material [19].

Silva and collaborators used five hemispherical-headed punches with a radius ranging from 4 mm to 25 mm to deform AA1050-H111 aluminum alloy in their research. The obtained results showed that the smaller the radius of the punch, the higher the stress concentration in the material, and the larger the radius of the punch, the easier it is to localize the material necking [20].

We have summarized studies in the field showing the influence of the diameter of the hemispherical punch used and the material of the semifinished parts used in incremental forming, presented in Table 2.

Another variant of punches used in the incremental forming process are punches that have a ball at the tip that can rotate during the incremental forming process, changing from sliding friction to rolling friction. Kim and his collaborators carried out a series of experiments to compare this type of punch with conventional ones. They observed an improvement in workpiece roughness and material deformability when the ball socket was used [29]. Other researchers, such as Lu and collaborators, have used an obliquely positioned ball indenter to reduce friction and improve surface quality [38].

Some researchers have also studied other types of tooling to perform the incremental forming process, such as the flat-headed punch, and analyzed its effect on the stresses and strains in the material. Ziran and collaborators compared two types of punches, hemispherical- and flat-headed. They noted an improvement in part accuracy and a reduction in the forces obtained when using flat-headed punches [30]. Lemopi Isidore and collaborators proposed the use of flat-headed punches to reduce the “pillow” effect (concave surface in the small base area) [39].

Eyckens and collaborators have used 5 to 10 mm diameter punches to study their influence in finite element analysis software [40]. Marques and collaborators have studied the influence of 8 to 12 mm diameter punches on the incremental forming of polymers [26].

Recently, in contrast to symmetrically shaped punches, Vanhove and collaborators used non-symmetrically elliptically shaped punches. They obtained an improvement in material deformability and part accuracy [41]. Abass compared the influence of punches with hemispherical, flat, or elliptical heads on incrementally deformed parts using finite element analysis software [42].

### 3.3. Lubrication Used in Incremental Forming

**Figure 6:**
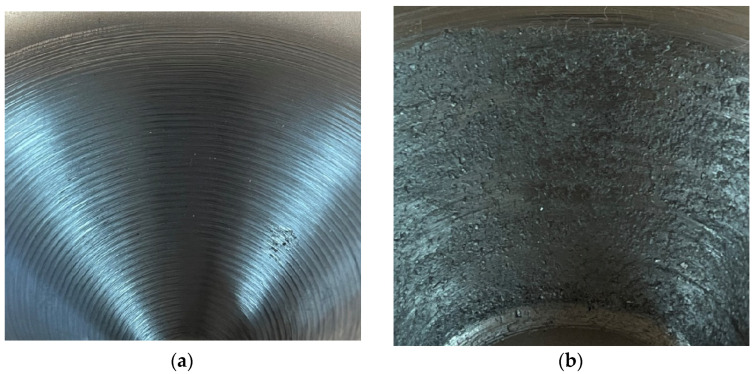
Comparison between the quality of a formed part (**a**) with lubricant and (**b**) without lubricant.

The area of contact between the punch and the blank is small, resulting in high contact pressure, which leads to increased frictional resistance. The friction between the punch and the blank has a large influence on the deformability of the material and the surface quality. To reduce the effects of friction, there are two solutions: the use of a punch with a ball and/or the use of lubrication. The use of a ball punch can improve the surface quality, as presented in Section 3.2. However, the friction between the punch and the blank can be reduced by the use of lubricants, and the surface quality can be improved considerably. Figure 6 shows the difference between a part where a lubricant has been used and a part where no lubricant has been used.

The use of lubricants is essential when two or more components come into contact in order to extend their service life by reducing friction and removing residual materials [43]. Lubricants have different characteristics, the most important being viscosity and density. In the field of classical plastic deformation processes, there is a lot of research on which lubricants can be used. The evaluation of the friction during these processes can be very complex, as can be seen in the research carried out by Figueiredo and collaborators [44]. Regarding the incremental forming process, the deformation mechanisms and the forces occurring during the process are different compared to classical plastic deformation processes [45]. There are few studies in the field on the influence of different lubricants on incrementally deformed parts.

Hussain and collaborators studied the influence of different lubricants on incrementally deformed titanium blanks [19]. Zhang and collaborators analyzed the influence of lubricants in the hot incremental forming process of aluminum alloys and noted that ceramic powder and solid graphite give remarkable results as well as a self-lubricating effect [46]. For low strength materials or low carbon steels, mineral oils are sufficient to achieve acceptable surface quality [29].

Najm and collaborators studied the influence of lubricants on the surface quality of AA1100 aluminum alloy semifinished products and noted that the use of a mineral oil has a positive effect on them [47]. Azevedo and collaborators analyzed the influence of five lubricants on parts made of AA1050 aluminum alloy and DP780 steel, and the results obtained are shown in Table 3 [48].

Syahrullail and collaborators suggested the use of a suitable additive to solve this problem [49]. Diabb and collaborators noted the high wear of aluminum blanks due to pieces becoming detached from the material [50]. Wan Nik and collaborators concluded that the rheology and dynamics of bio-based oils play an important role in improving heat distribution and waste disposal [51]. Unfortunately, most lubricants are petroleum-based and can cause irritation and allergies [52].

In the case of titanium blanks, Hussain and collaborators proposed the deposition of a porous film via a plasma electrolytic oxidation process in order to fix MoS_2_ powder particles (solid lubricant). They observed that using the proposed lubricant by this application method is effective for the studied process [53]. The same process of graphite oxide composite deposition was used by Mu and collaborators for Ti6Al4V alloy semifinished products, and the results showed that coated semifinished products have lower coefficient of friction and wear than uncoated semifinished products [54]. Table 4 summarizes the lubricants used by the researchers in incremental forming. This table provides a comprehensive overview of the various lubricants used in incremental forming processes, highlighting their application across a wide range of materials and part geometries. The table showcases how different lubricants, such as mineral oils, cutting oils, and solid powders (e.g., MoS_2_ or graphite), are utilized to optimize material deformability and surface quality.

For instance, Zhang [46] and Van Sy [55] employed advanced lubricants like nano-K_2_Ti_4_O_9_ and solid graphite powder for deforming AZ31 magnesium alloys into complex geometries like frustrum pyramids and cones, indicating the importance of lubricant choice in enhancing the process for lightweight materials. Similarly, Branker [56] demonstrated that even unconventional lubricants like used cooking oil can be effective in deforming aluminum alloys, suggesting that cost-effective alternatives may exist. The table also reveals the diversity in material applications, from aluminum alloys (e.g., AA5052, AA2024, AA6061) to high-strength materials like titanium alloys [66,67]. Lubricants like MoS_2_ and boron nitride sprays have been particularly effective for titanium alloys, where reducing friction and improving surface finish are critical.

Additionally, several researchers have explored the effect of lubricants on complex and specialized shapes, such as cranial implants [66] and prosthetic plates [68], showing the potential of lubricants in medical applications.

Overall, this table underscores the critical role lubricants play in incremental forming, influencing not only material behavior but also surface finish and part geometry. The variety of lubricants and materials explored highlights ongoing research aimed at optimizing incremental forming for both industrial and specialized applications.

### 3.4. Toolpaths Used for Incremental Forming of Tables

The path followed by the punch in the incremental forming process is the equivalent of a chipping operation, although in this case the tool does not remove the material, but locally deforms the material. The choice of toolpath has a huge impact on the accuracy of the part, surface quality, the deformability of the material and residual stresses in the material. The motion achieved by the punch is defined by the same parameters as for the chipping process. The feed rate is given by the speed at which the punch moves in contact with the blank and is measured in mm/min. The vertical step is the depth at which the punch enters the blank and is measured in mm. The rotational velocity is the speed at which the punch rotates about its own axis and is measured in rpm [25]. Research on the incremental forming process includes numerous comparative studies performed on simple geometries, such as frustrum cones, pyramids, or funnels [70]. These geometries are mainly characterized by the wall angle and depth of part. The generation of the punch toolpath starts by designing the parts in a CAD software, such as CATIA V5, followed by importing them into a CAM software that generates the toolpaths for the desired part [71].

**Figure 7:**
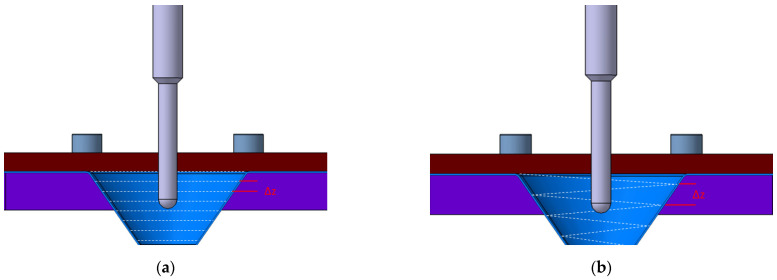
(**a**) In-plane contour curve type trajectory and (**b**) spiral-type toolpath.

Therefore, the choice of a toolpath must be performed considering these parameters. The most commonly used toolpaths in the field of incremental forming are in-plane contour curves followed by depth indentation or spiral movement. However, the spiral toolpath is difficult to implement for complex geometries. Figure 7 shows the two toolpaths, where ∆z is the vertical step and α is the part wall angle.

The in-plane contour curve toolpath can be further categorized according to the type of step with which the indentation is made, namely vertical and angular step. In the case of the vertical-step toolpath, the punch moves in the axial direction with a constant step (∆z) after each in-plane contour curve. Blaga and collaborators compared the influence of the vertical step with that of the angular step on the forces obtained during the incremental forming of DC04 steel blanks [72]. Skjoedt and collaborators presented a method to use this type of toolpath for more complex shaped parts as well [73]. The spiral toolpath is continuous and provides stability in terms of forming forces and better quality of machined surfaces [74]. In contrast, the other toolpath is discontinuous and worsens the quality of parts machined by incremental forming [75].

Jadhav noted the tendency of the sheet blank to twist when the spiral trajectory is used [76]. To solve this problem, Suresh and collaborators suggested the use of a bidirectional in-plane contour curve type toolpath, i.e., after each contour, the punch will move in the reverse direction [77]. Their results showed that the force values decreased significantly compared to the classical toolpath.

Another concern of researchers in the field has been the development of toolpath correction algorithms used in incremental forming to reduce dimensional errors due to springback of the material [78]. Some researchers have studied the influence of the type of toolpath on forming forces obtained during the process. Blaga and collaborators studied the influence of three strategies on the forming forces of DC04 steel blanks. The results showed that the minimum values are obtained when the in-plane contour curves-type toolpath is used [79]. Thibaud and collaborators studied the implementation of the two different toolpaths for the realization of a frustrum pyramid and showed that the in-plane contour curves toolpath leads to an improvement in the deformability of the material [80]. Arfa and collaborators studied the applicability of these toolpaths for the realization of a frustrum cone of AA3003—O alloy [74]. Liu and collaborators analyzed the influence of these toolpaths on the forming forces and noted that the in-plane contour curves toolpath has higher forces compared to the spiral toolpath [75]. Most of the researchers in the field have analyzed the influence of in-plane curves with spiral toolpaths in order to optimize the forming forces [80]. Table 5 shows some research in the field in which these comparisons and the observations made by the authors are presented.

The table presents a comparative analysis of various toolpath types used in the incremental forming process, focusing on their influence on forming forces and the success of part formation. Across multiple studies, a clear trend emerges: a spiral toolpath generally results in lower forming forces and more successful part manufacturing compared to an in-plane contour curve toolpath.

In summary, the table underscores the superiority of a spiral toolpath over an in-plane contour curve in incremental forming. The spiral toolpath not only lowers forming forces but also improves the chances of successful part-forming, especially for complex geometries. Additionally, tool speed emerges as a critical factor that can complement the toolpath choice to further optimize the process.

### 3.5. Materials Used in the Process

Many different materials have been used in the field of incremental forming, such as a wide variety of metals, polymers, or other types of materials with high deformability. Centeno and collaborators studied the deformability and the mode of failure occurrence in the aluminum alloy AlSi304—H111 when subjected to incremental forming by means of conventional tests: the uniaxial tensile test and Nakajima. Their aim was to verify the influence of the bending induced by the punch radius on the occurrence of material failure [81]. Ambrogio and collaborators analyzed the behavior of welded sheets under the incremental forming process. Their study focused on welded AA1050—O semifinished products with a thickness of 2 mm [82]. Serratore and collaborators showed that, by means of the welded sheets, the area where material thinning occurs can be controlled [83].

Piccininni and collaborators investigated the influence of technological parameters on the incremental forming of hot-formed titanium alloy semifinished products [84]. Ortiz et al. investigated the hot incremental forming of a 1.6 mm thick TI-6Al-4V titanium alloy. The results showed that high temperatures improve the deformability of the material and reduce the springback of the material [85].

Conte and collaborators studied the processing of composite plastics (PMC) and fiberglass-reinforced plastics (FRP) using finite element analysis software [86]. Bagudanch and collaborators analyzed the differences occurring in the SPIF and TPIF of polyvinyl chloride and polycarbonate semifinished products. The results showed that, in the case of the TPIF process, a higher accuracy and a reduction in the springback effect is obtained for both studied polymers [87].

Another area of interest for researchers in the field is the study of the process carried out on two-layer sheets. Hernández-Ávila and collaborators have studied the behavior of hot-welded polypropylene (PP) and Santoprene two-layer sheets. They observed a different behavior in this type of sheets, especially in the case of the polypropylene that is not in direct contact with the punch [88].

Palumbo and collaborators realized biodegradable magnesium medical prostheses coated with a thin PCL layer [89]. Zhang and collaborators performed incremental hot forming for 0.8 mm thick AZ31 magnesium alloy semifinished parts and focused on various methods of high-temperature lubrication [46]. Ambrogio and collaborators studied the deformability of magnesium alloy, AZ31, when the material is heated. Their results showed that the temperature and vertical step influence the deformability of this material, and the maximum wall angle is obtained when the incremental forming process is carried out at a temperature of 250 °C [90]. Table 6 shows the materials used by researchers in the field. In the research conducted by Silva [34], it was shown that PVC can be successfully deformed through incremental forming at room temperature, highlighting the adaptability of the process for polymer materials. Similarly, Davarpanah [91] explored the behavior of PLA, showing that it behaves similarly to metals, allowing the formation of complex geometries. The incremental forming process proves to be an excellent method for forming polymer materials like PVC and PLA, offering high flexibility in producing customized components without the need for expensive dies. This is particularly advantageous for industries that require low-volume production or rapid prototyping, where traditional molding or stamping processes might not be economically viable. Ben Said [92] focused on aluminum alloys, revealing that vertical step size has a significant impact on surface quality, while Najm [5] showed that smaller tool diameters improve the accuracy of incrementally formed aluminum parts. In the study by Mezher [93], a comparison between aluminum and steel sheets demonstrated that the maximum forming angles differ depending on the material, with DC04 steel achieving 75° and AA1050 aluminum 72°. Buffa [94] found that increasing the rotational speed during incremental forming raises the material’s temperature, improving formability, while Vahdani [66] highlighted that the best material deformability was achieved with the smallest vertical step and highest feed rate. Finally, Toan [95] demonstrated that pre-heating magnesium alloys significantly enhances their deformability, emphasizing the role of temperature in the incremental forming process.

- The accuracy and quality of parts in incremental forming are influenced by several key parameters, including tool diameter, feed rate, vertical step size, spindle speed, and sheet material properties. These factors are detailed as follows: Tool diameter: Smaller tool diameters generally produce parts with higher geometric accuracy due to the localized deformation. However, they increase the risk of the material thinning and cracking. Experimental studies show that using a 5 mm diameter tool results in higher precision, but material thinning exceeds 20%, while a 10 mm diameter tool reduces thinning to approximately 10% at the cost of slightly lower accuracy.
- Feed rate: Higher feed rates can significantly reduce forming time, making the process more efficient. However, they can increase surface roughness and the likelihood of defects such as wrinkles. For instance, feed rates above 1500 mm/min have been observed to compromise surface quality, while the ones around 800 mm/min offer a balance between speed and part quality.
- Vertical step size: This parameter determines the incremental depth of deformation per tool pass. Smaller step sizes (e.g., 0.2 mm) enhance surface finish and reduce springback, while larger step sizes (e.g., 1 mm) accelerate the process but may compromise dimensional accuracy.
- Spindle speed: Higher spindle speeds contribute to better lubrication distribution and surface finish but may increase frictional heat, potentially altering material properties. Optimal speeds vary depending on the material, with aluminum alloys performing best at speeds between 2000 and 3000 RPM.
- Sheet properties: The choice of material significantly affects the process, but this choice depends more or less on the final product that needs to be manufactured. Higher ductility materials, such as steel and copper, are more suitable for incremental forming due to their high formability, whereas lower ductility materials like titanium and aluminum require special lubricants or preheating to prevent cracking.

## 4. Dimensional Accuracy of Incrementally Formed Parts

**Figure 8:**
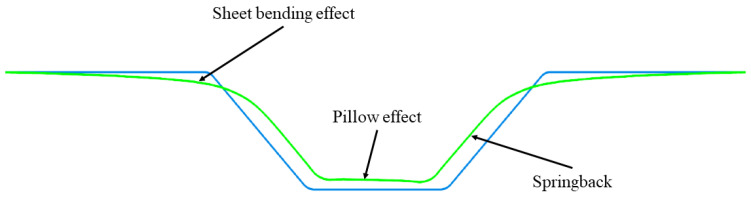
Representation of the most common defects of the incremental forming process.

The incremental forming process is more suitable for prototype production than conventional cold plastic forming processes. The process parameters used can affect the surface quality of the machined part as well as its dimensional and shape accuracy and can lead to material failure during manufacturing. In terms of part accuracy, the following flaws can be observed in the radius between the part wall and the flange, namely sheet-bending defect, springback of the material, the “pillow” effect, material thinning, and dimensional deviations. Acceptable geometric tolerances for industrial applications are in the range of ±0.5 mm [96] and in special cases can be ±0.2 mm [97]. The geometrical deviations resulting from the incremental forming process have been defined by Micari and collaborators as the deviations between the desired and the obtained shape [98]. During the process, as in any plastic deformation process, the undesirable phenomenon of springback of the material occurs [99]. Figure 8 shows some of the mentioned defects.

### 4.1. Material Springback

One of the main defects affecting the dimensional and shape accuracy of incrementally formed parts is material springback. This defect can be divided into two categories: the first is local springback, which occurs in the area near the punch, and the second is global springback, which occurs after the punch is removed from the sheet metal or after the sheet is removed from the fixing elements [100].

Researchers have given special attention to springback as it represents the main drawback of the process. Bambach and collaborators proposed to reduce springback by using a multi-pass toolpath which improves the accuracy of the final part [99]. Springback can be influenced by various factors, such as the shape and size of the punch, rotational speed, feed rate, sheet blank thickness, and residual stresses. Building on this, Yang Bingqian’s research introduces a novel predictive model using machine learning techniques like ong Short Term Memory (LSTM) and Support Vector Machine (SVM) regression to address springback. The model demonstrated a high accuracy with a “Coefficient of Determination” (R^2^) value of 0.9181, offering a robust solution for improving SPIF precision [101].

Wei and collaborators concluded that material springback has a significant impact on the accuracy of the final part shape and that there is a relationship between the wall angle of the part and the degree of springback [102]. Sun and collaborators studied the effect of ultrasonic vibration on the springback and surface quality of AA5052 aluminum alloy parts. Their results showed that using vibration in the incremental forming process reduces springback [103]. This incremental forming method has also been used by Zhang, and their results show a reduction in springback value and an improvement in dimensional accuracy due to the use of vibration [104]. Akrichi’s research introduces an experimental study for predicting the quality of parts formed through SPIF, including key factors like surface roughness, thickness, and springback. Additionally, the study adds circularity and position errors to the list of quality indicators. Using a modified backpropagation algorithm within an Artificial Neural Network (ANN), the study achieves more than 96.74% accuracy. The research highlights the influence of tool path strategies, spindle speed, incremental step size, and forming angle on the accuracy of complex geometries like double frustrum cones [105].

Recently, artificial neural networks have been used to control the process and develop efficient prediction methods. Han and collaborators utilized this method to predict springback using the finite element method. Analytical results were compared with experimental results, and these were close [106]. Wang and collaborators proposed a framework based on geometry/process-integrated Graph Neural Networks (GNN) for simulating axial springback in mesh-based metal bending. This framework, which employs an encode-process-decode structure, achieves accuracy comparable to the finite element method, with significant improvements in computational efficiency, demonstrating its potential for real-time springback simulation and optimization of bent tube quality [107].

### 4.2. The “Pillow” Effect of Incrementally Formed Parts

The “pillow” effect is a concave surface that occurs in the area of the small base of the parts, i.e., in the undeformed zone [108]. Many researchers have attempted to eliminate this defect in incremental forming by various methods. Among the first researchers to study this deviation were Ambrogio and collaborators. They observed that this deviation is strongly influenced by the shape of the punch tip, the depth and angle of the part wall, and the thickness of the sheet blank [109]. Micari and collaborators presented three types of deviations from the accuracy of incrementally deformed parts: springback in the wall area, the “pillow” effect in the small base area and the sheet bending defect [98]. Isidore and collaborators used finite element analysis to analyze the influence of the shape and size of the chamfer on this deviation when producing AA1050 aluminum alloy blanks. The results show that hemispherical chamfers accentuate this deviation and straight chamfers reduce it [39]. This effect is more visible for thin sheets deformed at an as large as possible wall angle.

Zhang and collaborators noticed a reduction in this defect when using TPIF [110]. Al-Ghamdi and Hussain studied the influence of the mechanical properties of the material on the “pillow” effect and observed that they do not affect this deviation [60]. Their results showed that this deviation increases with the height of the deformed part. Afzal noted in his research that a possible cause of the occurrence of this defect is due to the elastic behavior of the material in the small base area [111].

### 4.3. Material Thickness

Uneven distribution of material thickness during the incremental forming process is a common defect. Researchers in the field have studied many methods to improve this effect. Among the methods analyzed over the years is the use of a multi-pass toolpath. Li and collaborators presented an equation that helps to determine the number of passes for a toolpath, and research results have shown that the incremental forming process with a conjugate active die achieves the best material thinness distribution [112]. Zhang and collaborators analyzed the thickness distribution of 2A12—O aluminum alloy when a multi-pass toolpath is used [110]. Zhu and collaborators developed a new type of toolpath that leads to a uniform thickness distribution of AA1060 aluminum alloy [113]. Their method is based on the use of parallel planes for deforming the material so as to minimize the material stretch.

Ambrogio and collaborators used finite element analysis to determine the influence of technological parameters on the material thickness distribution for pure aluminum and aluminum alloy AA1050—O [82]. Using the same method, Salem and collaborators studied the deformability and thickness distribution of aluminum alloy AA7075—O [114]. Their research showed three different areas of incrementally deformed parts: the sheet bending zone, the thinning zone, and the steady-state zone. Azouzi and Lebaal optimized the finite element analysis used by means of response surface method and sequential algorithms. They improved the thickness distribution of the studied aluminum alloys by 7% by optimizing the process parameters and the punch toolpath [115].

## 5. Applications of the Incremental Forming Process

The incremental forming process offers the possibility to manufacture prototypes without the use of a die. The machining time is too long to be considered suitable for mass production. The cost of producing a part by incremental forming is difficult to estimate. The parts have to be designed in a CAD software and then the toolpath has to be generated in a CAM software, which can take several hours, depending on the complexity of the part. Therefore, parts made by this process should be classified as high value and low quantity.

The applications of the incremental forming process are significant in a variety of industries. This process is used in the manufacture of metal components, plastic structures and in the production of medical implants and electronic devices. It is also applied in the construction industry and in the production of metal structures, as well as in the oil and gas industry. In recent years, incremental forming has gained prominence due to its ability to produce customized and complex parts efficiently. Below are notable examples of its applications:

1. In the automotive and aerospace industry
  - Manufacture of metal components: Car bodies and airplane wings can be manufactured by incremental forming. This process provides lightweight but strong structures with complex shapes. This reduces vehicle weight and improves their performance. Jeswiet and his collaborators have developed prototypes specific to the car manufacturing industry for exhaust guarding [3]. Recent advancements include the use of incremental forming to produce lightweight panels for electric vehicles, contributing to energy efficiency. In aerospace, titanium components for aircraft interiors have been successfully produced, combining high strength with reduced material waste.
2. In the plastics and electronics industry
  - Manufacturing plastic components: The incremental forming process can be used to produce plastic objects with complex shapes, such as electronic housings, customized components, and other specific items.
3. In the medical industry
  - Production of implants and prostheses: The incremental forming process can be used to manufacture customized implants and individually adapted medical prostheses. Recent examples include cranial and maxillofacial implants tailored to specific patient anatomies, significantly reducing production time and improving patient outcomes.
4. In micro-electro-mechanical technology (MEMS)
  - Sensor manufacturing: The incremental forming process can be used in the production of MEMS sensors, such as accelerometers and gyroscopes. This enables the production of microscopic, accurate, and high-performance elements used in advanced electronic devices.
5. In the construction and oil-gas industries
  - Fabrication of metal structures: Incremental forming of welded plates is applied in the construction industry for the production of structural elements used in bridges, buildings, and marine structures. It provides strength and durability under static and dynamic loading.
  - Oil and gas equipment: The incremental forming process is used in the manufacture of storage tanks, pipelines, and marine structures used in the oil and gas industry. It provides resistance to pressure and aggressive environments, contributing to the safety of oil and gas systems.

**Figure 9:**
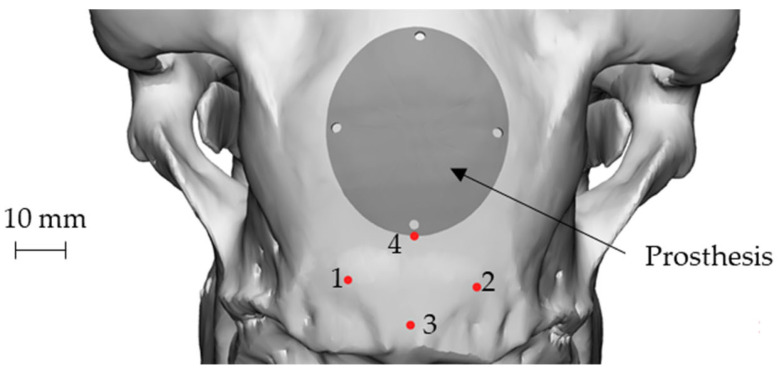
Prototypes of cranial implants for sheep created using the incremental forming process include four key landmarks and representations of both the defect and the prosthetic geometry: the attachment points for the nuchal tendons (points 1 and 2) and the sagittal plane (points 3 and 4) [116].

These are just a few examples of applications of the incremental forming process. The flexibility and benefits of this process make it valuable in various fields, helping to produce components and products with superior performance. The process is particularly valuable for industries that rely on rapid prototyping, low-volume production, and highly customized components. Figure 9 provides a visual representation of some of the key applications of the incremental forming process.

## 6. Sustainability of the Process

While it is widely accepted that incremental sheet forming offers greater flexibility, lower costs and shorter part-production times compared to traditional production methods, there is a lack of comprehensive sustainability assessments for this process in the literature. Nevertheless, a few authors have addressed this issue.

In paper [117], a comparison is made in terms of sustainability between the incremental forming process and the stamping process. The research has shown that the deformation energy is higher for incremental forming as compared to stamping, but incremental forming allows for some material savings.

Paper [118] presents a sustainability analysis focused on energy consumption (tackling topics like required deformation energy, electrical energy consumption, and comparison with the energy consumed for the stamping process) and on environmental impact assessment (tackling topics like CO_2_ emissions, optimal batch size, and material savings). As future directions to reduce energy consumption, the development of energy savings strategies by reducing the idle time and designing high-efficiency technological equipment for the process is recommended.

The research presented in [119] analyses the energy consumption of two forming processes, a conventional stamping process and an incremental deformation process, manufacturing through the two processes a part with the same geometry from a material with the same characteristics. As shown in this study, the manufacturing time is longer in the case of incremental deformation, which implies higher energy consumption; however, energy consumption can be reduced by reducing the incremental step. Research has also addressed the environmental impact of developing a product through these two processes. Energy consumption, tooling consumption, resulting sheet metal waste, recycling possibilities and estimation of the carbon footprint caused by the incremental forming process were considered. While incremental forming offers energy savings in comparison to stamping and provides the flexibility to produce customized components, it is essential to consider the extended production time that this process entails.

The performance of the incremental forming process in terms of required power, energy consumption, cost, CO_2_ emissions, processing time, and waste/material utilization is analyzed in [120]. The study concludes that incremental forming is a more sustainable process compared to conventional forming methods, especially for small production volumes (prototypes). By optimizing parameters such as lower spindle speeds, higher feed rates and larger step sizes, the sustainability of the process can be further improved. In addition, as other studies have shown, optimized tool paths and the development of energy-efficient machines are expected to further improve process performance.

In [121], a process planning strategy is proposed (which includes the selection of parameters and their use to estimate the necessary compensations for accuracy improvements, the choice of component orientation, tool type, and support force) to process parts through double-side incremental forming. The results indicated that applying this strategy can reduce process energy consumption by 10 to 50%.

## 7. Conclusions

In this article, we have presented a state-of-the-art insight of the recent research conducted in the field of the incremental forming process and the technology used to implement it. Through a thorough review of the literature, we have highlighted the contributions and challenges encountered in the development of this process, showcasing its potential across various industries, particularly in automotive manufacturing. Researchers have contributed significantly to the widespread implementation of incremental forming by focusing on improving process efficiency, dimensional accuracy, and surface quality.

The flexibility and adaptability of incremental forming is particularly highlighted in SPIF, which is widely used due to its ease of implementation and its ability to produce a variety of geometries without the need for expensive, specialized dies. However, TPIF and incremental forming with conjugated active die (IFCAD) also offer significant advantages for improving dimensional accuracy and enabling the formation of more complex geometries.

**Figure 10:**
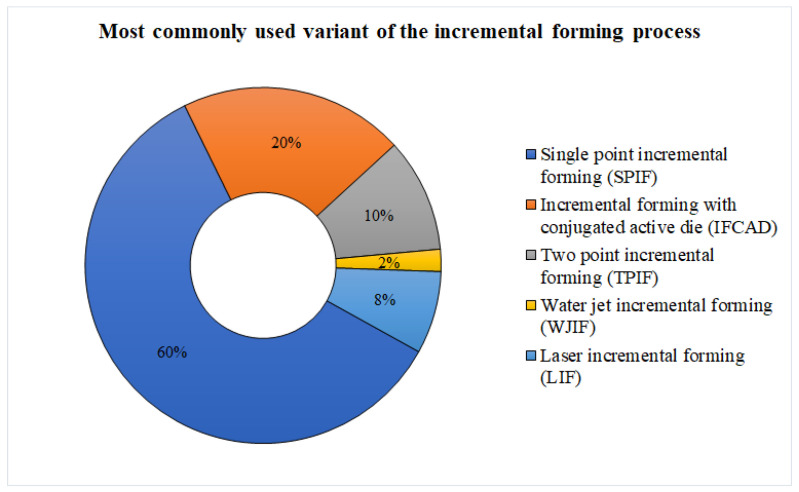
Degree of use of incremental forming process variants.

As shown in Figure 10, SPIF dominates the field due to its high flexibility and cost-effectiveness, allowing for rapid prototyping and small-series production. However, TPIF and IFCAD are gaining traction for specific applications that require improved precision and control over the deformation forces, offering better dimensional accuracy and surface quality in more demanding industrial applications.

Despite these advances, the accuracy of the formed parts remains a key challenge, and further research is required to overcome the issues of low dimensional accuracy, surface quality, material thinning, and defects such as springback and sheet bending. These challenges must be addressed to enhance the application of incremental forming for high-volume production.

To overcome the current limitations, future research should focus on process optimization, including improving toolpath strategies and adjusting material-specific parameters to enhance accuracy and reduce defects. Special attention must also be given to new mathematical models that, by considering process parameters, predict the final shape of the part, evaluate geometric deviations, and provide input data to compensate the toolpath, thereby reducing low precision to an industry-accepted minimum. Additionally, incremental forming should be expanded to include high-strength alloys, composite materials, and plastics, using customized tooling and lubrication strategies. The development of advanced equipment, such as specialized CNC machines and robotic systems, will improve precision, while integrating real-time monitoring and artificial intelligence will optimize process parameters, reducing defects and waste. Automation and scalability are essential for adapting the process to high-volume production while maintaining precision. Also, future research should address energy efficiency, utilizing bio-based lubricants and improving material recycling. Moreover, the industrial applications of incremental forming are expanding, with significant advancements in the automotive, aerospace, and medical sectors, and combining this process with additive manufacturing opens new possibilities for producing customized and complex parts.

In conclusion, incremental forming offers significant promise for next-generation manufacturing, especially for customized and complex geometries. However, its future success depends on solving challenges related to accuracy, process efficiency, and scalability. With continuous advancements in tooling, real-time monitoring, and automation, incremental forming can become a sustainable, versatile, and cost-effective alternative to traditional forming processes, ready to meet the demands of modern industries.

## Figures and Tables

**Table 1:** Comparison of Incremental Forming Methods: Characteristics, Applications, Advantages, and Disadvantages.

Method	Description	Applications	Advantages	Disadvantages
Single Point Incremental Forming (SPIF)	Single hemispherical or parabolic tool deforms the sheet incrementally. Suitable for low-volume production and prototyping.	Prototyping, low-volume production	Simple setup, high flexibility	Reduced accuracy
Two Point Incremental Forming (TPIF)	Includes a supporting tool or counterpunch to enhance precision and minimize springback. Suitable for high-precision components.	Aerospace, medical devices	Improved accuracy	More complex tooling
Incremental Forming with Conjugated Active Plate (IFCAP)	Uses an active die conjugated to the part geometry for enhanced control and surface quality. Ideal for complex geometries.	Complex geometries, precision parts	High precision, surface quality	Higher cost, complex setup

**Table 2:** Punch sizes used in the literature.

Study	Tool Diameter [mm]	Material	Conclusions
Ham [18]	4.7625 and 12.7	AA3003—O	Reducing the tool diameter increases the material deformability
Ham [21]	9.525, 12.7, and 19.05	AA6451, AA5182, and AA5754	
Hussain [22]	8, 12, and 16	Titanium	
Martins [23]	10 and 15	POM, PE, PA, PVC, PC	
Petek [24]	10 and 16	DC05	
Durante [25]	5, 10, and 15	AA7075—O	
Silva [20]	8, 12, 20, 30, and 50	AA1050—H111	
Marques [26]	8, 10, and 12	PET, PA, PVC, and PC	
Shanmuganatan [27]	2.5, 5, and 10	AA3003—O	
Centeno [28]	6, 10, and 20	SS304	
Kim [29]	5, 10, and 15	AA1050—O	Using an optimal tool diameter improves material deformability
Ziran [30]	4, 6, and 10	AA3003—O	
Hussain [19]	6, 8, 11, 14, and 16	AA2024—O	
Al-Ghamdi [31]	2.2, 3.6, 4.4, 5.4, 6.6, and 7.8	AA2024—O, AA2024—T6, AA1060—O, AA1060—H24, AA5083—O, DS Steel, and Cu H59	
Strano [32]	2.2, 3, and 6.4	AA1050—O	Increasing the tool diameter improves material deformability
Frazen [33]	10 and 15	PVC	
Silva [34]	10 and 15	PVC	
Li [35]	10, 20, 24.5, and 30	AA7075—O	
Golabi [36]	6 and 14	SS304	
Bagudanch [37]	6 and 10	PVC	

**Table 3:** Influence of lubricants on parts made of AA1050 and DP780 steel [48].

Lubricant	Type of Lubricant	Viscosity at 40 °C [mm^2^/s]	Density [kg/L]	Melting Point [°C]	Effect on: AA1050	Effect on: DP780
Repsol SAE 30	Mineral oil	105	0.884	215	**△**	▽
Total Finarol B 5746	Mineral oil	9.75	0.904	150	▽	**△**
Moly Slip AS 40	Paste	N/A	1.76	190	▽	**△**
Weicon AL-M	Paste	185	0.92	N/A	**△**	▽
Moly Slip HSB	Paste	N/A	N/A	195	—	—

Legend: **△**: Positive effect on the material’s forming process. ▽: Negative effect on the material’s forming process. —: No significant effect observed.

**Table 4:** Lubricants used in the specialized literature.

Author	Lubricant	Material	Shape of Deformed Part
Zhang [46]	nano-K_2_Ti_4_O_9_ and organic binder	AZ31	Truncated pyramid
Van Sy [55]	Solid graphite powder, MoS_2_, MLS_2_	AZ31	Truncated cone
Branker [56]	75W-140 transmission oil, used cooking oil	AA3003—O	Hat-shaped part
Ingarao [57]	Mineral oil	AA5754	Truncated cone and truncated pyramid
Loganathan [58]	SAE 20W-40	AA6061	Truncated cone
Devarajan [59]	Cutting oil	AA2024	Complex parts
Al-Ghamdi [60]	Mineral oil	AA2024—T4	Truncated cone
Trzepieciński [61]	SAE 75W—85 transmission oil	AA2024—T3	Stiffening ribs
Riaz [62]	Mineral oil	AA2219—O, AA2219—T6	Truncated pyramid
Mugendiran [63,64]	Motor oil	AA5052	Truncated pyramid, truncated cone with a 70° wall angle, and truncated cone with variable wall angle
Kumar [65]	Alpha SP68, Alpha SP150, Alpha SP320	AA6063—O	Truncated cone
Vahdani [66]	Solid graphite powder, boron nitride spray ROCOL RTD, vegetable oils	Ti—6Al—4V	Truncated cone and cranial implant shape
Lu [67]	MoS_2_	Pure titanium	Truncated cone
Sbayti [68]	Chlorine-containing oils	Grade 1 titanium	Prosthetic plate
Vanhove [69]	Nuto 46 hydraulic oil, WEICON ASW 040P ceramic lubricant MoS_2_	Grade 2 titanium	Clavicle, facial implant, and hemispherical shape

**Table 5:** Trajectories used by researchers.

Authors	Trajectory	Material	Shape of Part	Results
Blaga [79]	In-plane contour curve toolpath compared to spiral toolpath	DC04 steel	Frustrum cone with a diameter of 44 mm, depth of 12 mm, and a wall angle of 55°	Axial force is lower with the spiral toolpath
Thibaud [80]	In-plane contour curve toolpath compared to spiral toolpath with 1° angular step	FPG copper alloy	Frustrum pyramid with a side of 6 mm, depth of 4 mm, and a wall angle of 25°	The part was successfully formed with the spiral toolpath, but broke before reaching the desired depth with the in-plane contour curve toolpath
Arfa [74]	In-plane contour curve toolpath compared to spiral toolpath	AA3003-O aluminum alloy	Frustrum pyramid with a side of 180 mm and wall angle of 50°, frustrum cone with a large base diameter of 180 mm and depth of 40 mm	Forming forces were smaller with the spiral toolpath compared to the in-plane contour curve toolpath
Durante [25]	In-plane contour curve toolpath compared to bidirectional toolpath	AA3003-T0 aluminum alloy	Frustrum pyramid with a wall angle of 60°	Forming forces decreased as the tool rotation speed increased

**Table 6:** Materials used by the researchers.

Author	Material	Parameters used		Shape of part	Observations
Silva [34]	PVC	Sheet thickness:	2 mm and 3 mm	Frustrum cone	PVC blanks can be successfully deformed through incremental forming at room temperature.
		Tool diameter:	10 mm and 15 mm		
		Vertical step:	0.5 mm		
		Feed rate:	1500 mm/min		
Davarpanah [91]	PLA	Sheet thickness:	0.7 mm	Frustrum cone with wall angle varying from 30° to 90°; Frustrum cone with wall angle of 55°, 65°, and 75°	Same behavior as in metals was observed.
		Tool diameter:	5 mm		
		Vertical step:	0.2 mm, 0.4 mm, 0.6 mm, 0.8 mm, and 1 mm		
		Feed rate:	300 mm/min		
Ben Said [92]	AA1060-H14 aluminum alloy	Sheet thickness:	0.6 mm	Hemispherical shape	Vertical step size affects surface quality.
		Tool diameter:	10 mm		
		Vertical step:	0.1 mm and 0.2 mm		
		Feed rate:	3.75 mm/min		
		Spindle speed:	75 rpm		
Najm [5]	AlMn1Mg1 aluminum alloy	Sheet thickness:	0.22 mm	Truncated cone	Research shows that tool diameter influences the accuracy of incrementally formed parts.
		Tool diameter:	2 mm, 4 mm, and 6 mm		
		Vertical step:	0.05 mm		
		Feed rate:	1500 mm/min		
		Spindle speed:	2000 rpm		
Mezher [93]	AA1050 aluminum alloy and DC04 steel	Sheet thickness:	1 mm	Frustrum cone with wall angle of 45°, 55°, 65°, 70°, and 75°	Maximum forming angle is 75° for DC04 and 72° for AA1050.
		Tool diameter:	16 mm		
		Vertical step:	0.05 mm		
		Feed rate:	600 mm/min		
		Spindle speed:	450 rpm		
Buffa [94]	Aluminum alloys: AA1050-O, AA1050-H24, AA6082-T6	Sheet thickness:	1 mm	Frustrum cone	Higher rotational speeds lead to increased material temperature.
		Tool diameter:	12 mm		
		Vertical step:	1 mm		
		Feed rate:	2000 mm/min		
		Spindle speed:	100–1000 rpm		
Vahdani [66]	Ti-6Al-4V titanium alloy, AA6061 aluminum alloy, and DC01 steel	Sheet thickness:	1 mm	Frustrum cone	Best material deformability was achieved with the smallest vertical step and the highest feed rate.
		Tool diameter:	10 mm		
		Vertical step:	0.2 mm and 0.3 mm		
		Feed rate:	400 mm/min and 900 mm/min		
Toan [95]	AZ31B-H24 magnesium alloy	Sheet thickness:	1 mm	Frustrum pyramid	Heating the parts leads to better deformability.
		Tool diameter:	6 mm		
		Vertical step:	0.4 mm		
		Feed rate:	400 mm/min		
		Spindle speed:	3000 rpm and 4000 rpm		

## Data Availability

No new data were created or analyzed in this study.
